# Pre-existing chronic kidney disease, aetiology of acute kidney injury and infection do not affect renal outcome and mortality

**DOI:** 10.1007/s40620-023-01774-x

**Published:** 2023-10-03

**Authors:** Anna Buckenmayer, Nadja Siebler, Christian S. Haas

**Affiliations:** Department of Internal Medicine, Nephrology and Intensive Care Medicine, Phillips University, Baldinger Straße 1, 35043 Marburg, Germany

**Keywords:** Acute kidney injury, Aetiology, Infections, Outcome, Chronic kidney disease

## Abstract

**Background:**

We aimed to study the role of aetiology, pre-existing chronic kidney disease (CKD) and infections in acute kidney injury (AKI) on renal outcome and mortality.

**Methods:**

This retrospective study analysed patients with AKI admitted to a university nephrology department from January 1st, 2020 through December 31st, 2020. Aetiology of AKI, underlying renal disease in case of pre-existing CKD and presence of infections were assessed. Development of renal function and risk of death were studied with follow-up until January 31st, 2023.

**Results:**

Of 1402 patients screened, 432 patients (30.8%, 67.9 ± 15.4 years) fulfilled the inclusion criteria, half of the population presented with advanced CKD. Even though CKD patients were more often in need of chronic dialysis at time of discharge (6.9% vs 4.5%, *p* < .001), duration of hospital stay was shorter and in-hospital mortality tended to be lower when compared to AKI without prior renal disease. Neither aetiology of AKI nor pre-existing CKD had an impact on the combined endpoint of end-stage kidney disease and mortality (log rank 0.433 and 0.909). Overall, septic patients showed the highest in-hospital mortality (23.5%) and longest hospital stay (30.0 ± 22.8 days, *p* < .001), while patients with urosepsis had the shortest hospitalisation time (9.7 days) with lowest risk for dialysis (4.4%). Of note, outcome did not differ in patients with AKI when considering the infectious status.

**Conclusions:**

Overall renal outcome and mortality in AKI patients were not affected by the cause of AKI, pre-existent CKD or infectious status. Only severity of AKI had a negative impact on outcome.

**Graphical abstract:**

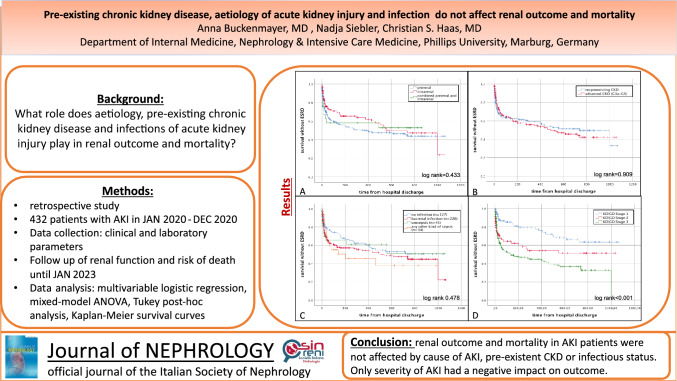

**Supplementary Information:**

The online version contains supplementary material available at 10.1007/s40620-023-01774-x.

## Introduction

Acute kidney injury (AKI) is a common problem in hospitalised patients. In fact, one of five in-patients experiences AKI, while patients on intensive care units are even more affected [1, 2]. Acute kidney injury is defined as a sudden decline in kidney function, classified into three stages, most commonly using the AKIN or—more recently—the KDIGO criteria [3]. Being associated with prolonged hospital stay and increased risk of permanent renal replacement therapy (RRT), AKI has a relevant impact on morbidity. In addition, it has repeatedly been demonstrated that AKI is an independent, significant risk factor for short- and long-term mortality [4–6]. Acute kidney injury itself is a complex entity with various underlying pathogenic mechanisms. For practical purposes, it is generally divided into three categories: (1) AKI with prerenal cause and renal hypoperfusion due to hypovolemia or congestive heart failure; (2) intrinsic AKI as the result of structural and/or functional damage to the kidney itself; and (3) postrenal injury attributed to urinary tract obstruction [7]. While the latter category can be easily identified by ultrasound with a straight-forward therapeutic approach by reinstating urinary drainage, all other causes require a thorough diagnostic analysis and target-specific therapy.

In daily clinical practice, the simultaneous presence of prerenal and intrinsic AKI is common and often the result of competing factors, such as exposure to contrast medium, nephrotoxic medications, hypotension, and infection. Some underlying causes may affect kidney function even through several mechanisms: e.g., infections, which can result in impaired renal blood flow, acute tubular necrosis and the release of pro-inflammatory mediators, all resulting in renal damage [8, 9]. Of note, more than 40% of patients with AKI already present with pre-existing chronic kidney disease (CKD) [10]. Reasons for CKD are heterogeneous, and include renal involvement in hypertension, diabetes, vasculitis or primary glomerulonephritis [11]. The role of the underlying cause of CKD in AKI though has not yet been investigated with respect to renal and patient outcome. Furthermore, the impact of pre-existing CKD and the underlying renal disease on renal recovery and patient mortality remains unclear.

The objective of the present study was: (1) to differentiate outcome of AKI with respect to the underlying aetiology of renal impairment and the potential role of infections; and (2) to identify the impact of pre-existing CKD on all-cause mortality and renal outcome.

## Materials and methods

We performed a retrospective cross-sectional, descriptive cohort study at the Department of Internal Medicine, Nephrology and Intensive Care Medicine at the University Hospital in Marburg, Germany. All in-patients admitted between January 1st, 2020 and December 31st, 2020 were screened for the presence of AKI using the electronic patient record system. Patients treated on the nephrology ward with an ICD code for all forms of acute kidney injury (ICD-10 N17.01–N17.99) were identified. Exclusion criteria were age < 18 years and patients with a functional kidney transplant.

After identifying eligible patients, AKI stages were checked for each individual using the KDIGO definition; in addition, the presence of pre-existing CKD was determined [3]. Aetiology of AKI, the underlying renal disease in case of pre-existing CKD and duration of hospital stay were assessed from the patients’ charts. The established diagnosis was the result of complex nephrology care supervised by an experienced senior nephrologist, considering medical history, clinical signs and symptoms, imaging as well as laboratory parameters. The diagnostic approach included differentiated proteinuria analysis, measurement of urinary concentration of electrolytes, creatinine, and osmolality in spot urine or/and 24 h urine collection, and serum parameters to differentiate type of AKI. When deemed necessary, a kidney biopsy had been performed. After discharge, patients were followed-up until January 31st, 2023 with respect to kidney function, development of end stage kidney disease (ESKD) and death using the patients’ electronic medical records.

**Fig. 1:**
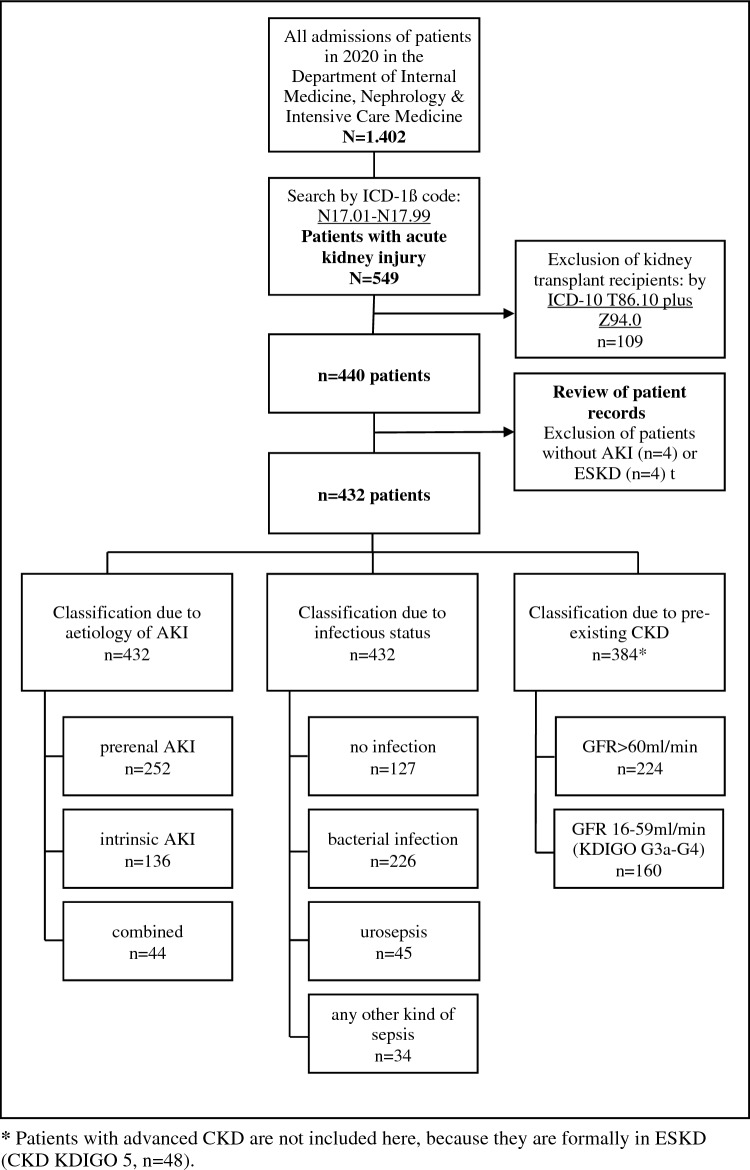
Selection and classification process of patients. *AKI* acute kidney injury, *RRT* renal replacement therapy, *CKD* chronic kidney disease, *ESKD* end-stage kidney disease

For subsequent data analysis, all patients were classified in three different ways: (1) grouping regarding aetiology of AKI (prerenal, intrinsic, combined); patients with isolated postrenal AKI were excluded; (2) categorisation with respect to infectious status (no infection, bacterial non-systemic infection, urosepsis and any other kind of sepsis); and (3) classification with respect to pre-existing CKD (GFR > 60 ml/min vs. GFR16-59 ml/min, Fig. 1). Severe AKI was defined as AKI stage 3. Definition criteria for sepsis were based on the guidelines of the Society of Critical Care Medicine and the European Society of Intensive Care Medicine, updated in 2016 [12].

To identify potential risk factors for the development of severe AKI or death, multiple variables at the time of admission, including age, sex, serum sodium level, proteinuria, as well as urinary α1-microglobulin and albumin were checked, and a multivariable logistic regression calculation was performed with respect to intra-hospital death and RRT at time of discharge. Cox regression was used to retrospectively analyse the potential impact of type of AKI, pre-existing CKD and infectious status on outcome.

The primary combined endpoint was defined as death and/or development of ESKD in need of renal replacement therapy. Sample size calculations were performed prior to data collection to determine the number of patients needed to detect significant changes between groups. For this purpose, the G*Power calculator by Faul et al. was used [13], calculating for a mixed-model ANOVA with two, three and four groups, respectively. Effect size was set at a low level (*f* = 0.15), while defining high statistical power (0.95). Using these assumptions, calculations revealed a sample size of at least *n* = 160 patients with 40 patients per group (Supplemental Table 1). Potential risk factors for the development of severe AKI and its influence on outcome were assessed using a binominal logistic regression model. Statistical analysis was performed using SPSS statistics (version 28) and Excel (version 16.0). Descriptive data are presented as total count or mean, unless otherwise stated. One-way ANOVAs were conducted to assess differences between patient groups; where applicable, Tukey post-hoc analysis was subsequently performed. Renal impairment requiring permanent RRT in ESKD or death were imaged using Kaplan–Meier survival curves, with differences objectified by log-rank test. A two-tailed *p* value < 0.05 was considered statistically significant.

The study was given a waiver by the Ethics Committee, Philipps University, Marburg, Germany (11/2021 RS 21/88). The study and the manuscript comply with the STROBE checklist for observational studies [14], available in the Supplemental Table 2.

## Results

In total, 1402 patients were screened, among them, 459 had a diagnosis of AKI (Fig. 1), and 432 patients fulfilled the inclusion criteria. Average age was 67.9 ± 15.4 years with a male predominance (*n* = 272, 63.0%). One hundred seventy-three patients were treated in the ICU at some point. Chronic kidney disease was present prior to admission in more than half of the study population (n=235, 54.3%), with most patients having advanced CKD stages. Baseline characteristics of patients without CKD and with mild CKD, and those with advanced CKD stages (KDIGO G3a-4) are presented in Table 1: CKD patients were significantly older (74.0 vs. 65.7 years, *p* < 0.001) and more often in need of permanent RRT at the time of discharge (13.9% vs. 5.2%, *p* = 0.004).

**Table 1:** Baseline characteristics of patients comparing patients with GFR > 60 ml/min pre-admission (no CKD, CKD KDIGO G1, G2) with patients with pre-existing CKD (KDIGO G3a–G4)

	GFR > 60 ml/min (*n* = 224)	GFR 16–59 ml/min (*n* = 160)	*p* value
Age	65.7	74.0	** < 0.001**
Male	145 (64.7%)	93 (58.1%)	0.20
Duration of hospital stay (d)	15.8	12.5	**0.01**
Temporary dialysis	51 (22.8%)	30 (18.8%)	0.49
Discharge with dialysis	10 (4.5%)	11 (6.9%)	** < 0.001**
In-hospital mortality	48 (21.4%)	21 (13.1%)	0.08
Severity of AKI			
AKI I	54 (24.1%)	53 (33.1%)	0.15
AKI II	53 (23.7%)	35 (21.9%)	0.58
AKI III	117 (52.2%)	72 (45.0%)	0.29
Pre-existing CKD			
No CKD	191 (85.3%)	/	/
KDIGO G1	8 (3.6%)	/	/
KDIGO G2	25 (11.2%)	/	/
KDIGO G3a	/	39 (24.2%)	/
KDIGO G3b	/	58 (36.2%)	/
KDIGO G4	/	63 (39.3%)	/
Comorbidities			
Hypertension	161 (71.9%)	101 (63.1%)	0.16
Diabetes mellitus	69 (30.8%)	55 (34.4%)	**0.003**
Chronic heart failure	118 (52.7%)	71 (44.4%)	0.17
Laboratory parameters			
Pre-admission creatinine	1.06	1.60	** < 0.001**
First creatinine	2.59	2.99	** < 0.001**
Maximum creatinine	3.37	3.62	** < 0.001**
Last creatinine prior to dismissal^a^	1.64	2.11	** < 0.001**

Patients with advanced CKD, formally in ESKD (CKD KDIGO 5, *n* = 48) are not shown in this table

*AKI* acute kidney injury, *CKD* chronic kidney disease

^a^Discharged patients still in need of renal replacement therapy are excluded

Surprisingly, in-hospital mortality tended to be lower in the advanced CKD group (13.1% vs. 21.4%, *p* = 0.08) and length of hospital stay was shorter (12.5 vs. 15.8, *p* = 0.01). In about one third of the CKD patients, hypertensive and/or diabetic nephropathy was the underlying renal disease (37.0%), followed by cardiorenal and hepatorenal syndrome (12.0%) and primary or secondary glomerulonephritis (13.0%, Supplemental Fig. 1). Patients with IgA nephropathy were younger than the average CKD population (49.0 ± 15.8 years, *p* < 0.001), had the shortest length of hospital stay (9.0 ± 8.1 days, *p* = 0.025) and did not require temporary or permanent RRT. Of note, all other CKD patients had comparable demographic and baseline characteristics (Supplemental Table 3).

When considering AKI patients with respect to their infectious status (no infection, bacterial non-systemic infection, urosepsis and any other kind of sepsis, Table 2), patients with urosepsis showed the shortest hospitalisation time (9.7 days) and were least often in need of permanent RRT at the time of discharge (*n* = 2, 4.4%), even though groups were comparable with respect to severity of AKI. Patients with any other kind of sepsis predominantly presented with pulmonary sepsis (*n* = 14, 41.2%), while other septic foci were less common (skin: *n* = 6, 17.6%; abdominal: *n* = 5, 14.7%; infection of central venous catheters: *n* = 4, 11.8%; endocarditis: *n* = 2, 5.9%; not known: *n* = 3, 8.8%). Overall, septic patients showed the highest in-hospital mortality (23.5%) and longest hospital stay (30.0 ± 22.8 days, *p* < 0.001). When comparing patients with any kind of sepsis to patients without any infection, hospital stay was also significantly longer (18.5 vs. 10.4 days, *p* < 0.001) and risk of temporary dialysis was higher (29.1% vs. 15.7%, *p* = 0.02). There were no significant differences when comparing patients’ outcome with respect to aetiology of AKI (Supplemental Table 4).

**Table 2:** Baseline characteristics of AKI patients with respect to infectious status

	Total (*n* = 432)	Patients without infection (*n* = 127)	Bacterial infection (*n* = 226)	Urosepsis (*n* = 45)	Any other sepsis (*n* = 34)	*p* value
Age (years)	69.7	67.9	70.6	72.0	66.7	0.21
Male	272 (63.0%)	80 (63.0%)	133 (58.8%)	30 (66.7%)	29 (85.3%)	0.021
Duration of hospital stay (d)	15.0	10.3	16.4	9.7	30.0	** < 0.001**^**a**^
Temporary dialysis	103 (24.0%)	20 (15.9%)	60 (26.8%)	8 (17.8%)	15 (44.1%)	**0.003**^**b**^
Discharge with dialysis	39 (9.0%)	8 (6.3%)	26 (11.5%)	2 (4.4%)	3 (8.8%)	0.27
In-hospital mortality	76 (17.5%)	16 (12.7%)	43 (19.2%)	9 (20.0%)	8 (23.5%)	0.32
Severity AKI						
AKIN I	120 (27.8%)	33 (26.0%)	67 (29.6%)	18 (28.9%)	7 (20.6%)	.17
AKIN II	102 (23.6%)	37 (29.1%)	49 (21.7%)	11 (24.4%)	5 (14.7%)	.35
AKIN III	210 (48.6%)	57 (44.9%)	110 (48.7%)	21 (46.7%)	22 (64.7%)	.14
Pre-existing CKD	235 (54.3%)	84 (66.1%)	123 (54.4%)	12 (26.7%)	16 (47.0)	
KDIGO G1	8 (1.9%)	3 (2.4%)	3 (1.4%)	1 (2.2%)	1 (2.9%)	0.23
KDGIO G2	25 (5.9%)	10 (7.9%)	12 (5.4%)	2 (4.4.%)	1 (2.9%)	0.59
KDGIO G3a	39 (9,1%)	12 (9.4%)	23 (10.4%)	2 (4.4.%)	2 (5.9%)	0.19
KDIGO G3b	58 (13.6%)	23 (18.1%)	29 (13.1%)	0 (0.0%)	6 (17.6%)	0.43
KDIGO G4	62 (14.5%)	24 (18.9%)	34 (15.3%)	2 (4.4.%)	2 (5.9%)	0.36
KDIGO G5	43 (10.1%)	12 (9.4%)	22 (9.9%)	5 (11.1%)	4 (11.8%)	0.45
Comorbidities						
Hypertension	296 (68.5%)	83 (65.9%)	164 (73.9%)	19 (42.2%)	20 (60.6%)	0.20
Diabetes mellitus	149 (34.5%)	49 (38.9%)	69 (31.1%)	29 (64.4%)	12 (35.3%)	0.34
Chronic heart failure	209 (48.4%)	60 (47.6%)	113 (50.9%)	20 (44.4%)	16 (48.5%)	0.85
Laboratory parameters (mg/dl)						
First creatinine	3.12	3.16	3.06	3.40	2.91	0.84
Maximum creatinine	3.86	3.71	3.9	4.0	3.97	0.90
Last creatinine^c^	1.95	2.12	1.9	1.89	1.52	0.20

*AKI* acute kidney injury, *CKD* chronic kidney disease

^a^Any other kind of sepsis > no infection, bacterial infection, urosepsis; bacterial infection > no infection, urosepsis

^b^Any other kind of sepsis > no infection

^c^Discharged patients still in need of renal replacement therapy are excluded post-hoc comparisons between groups (Tukey’s test)

To identify potential risk factors for the development of severe AKI, a multivariable logistic regression for intra-hospital death and discharge in need of RRT showed no significance for most parameters. Proteinuria was the only identifiable risk factor for the permanent need for RRT at time of discharge (*p* = 0.004). Furthermore, neither type of AKI nor pre-existing CKD or infectious status seemed to have a prognostic impact on renal outcome and mortality (Supplemental Tables 5 and 6).

**Fig. 2:**
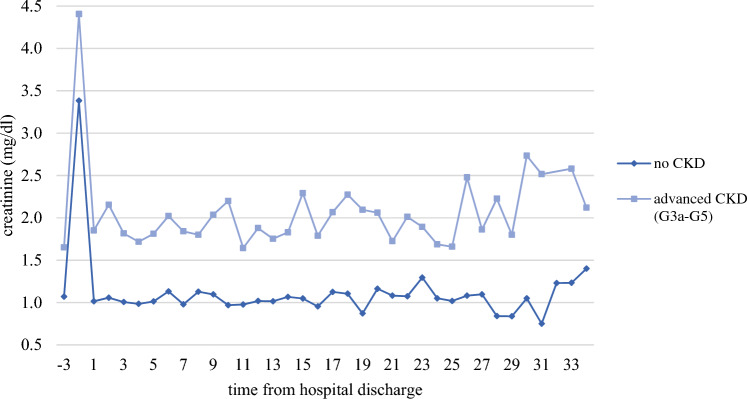
Follow-up of mean serum creatinine values

**Fig. 3:**
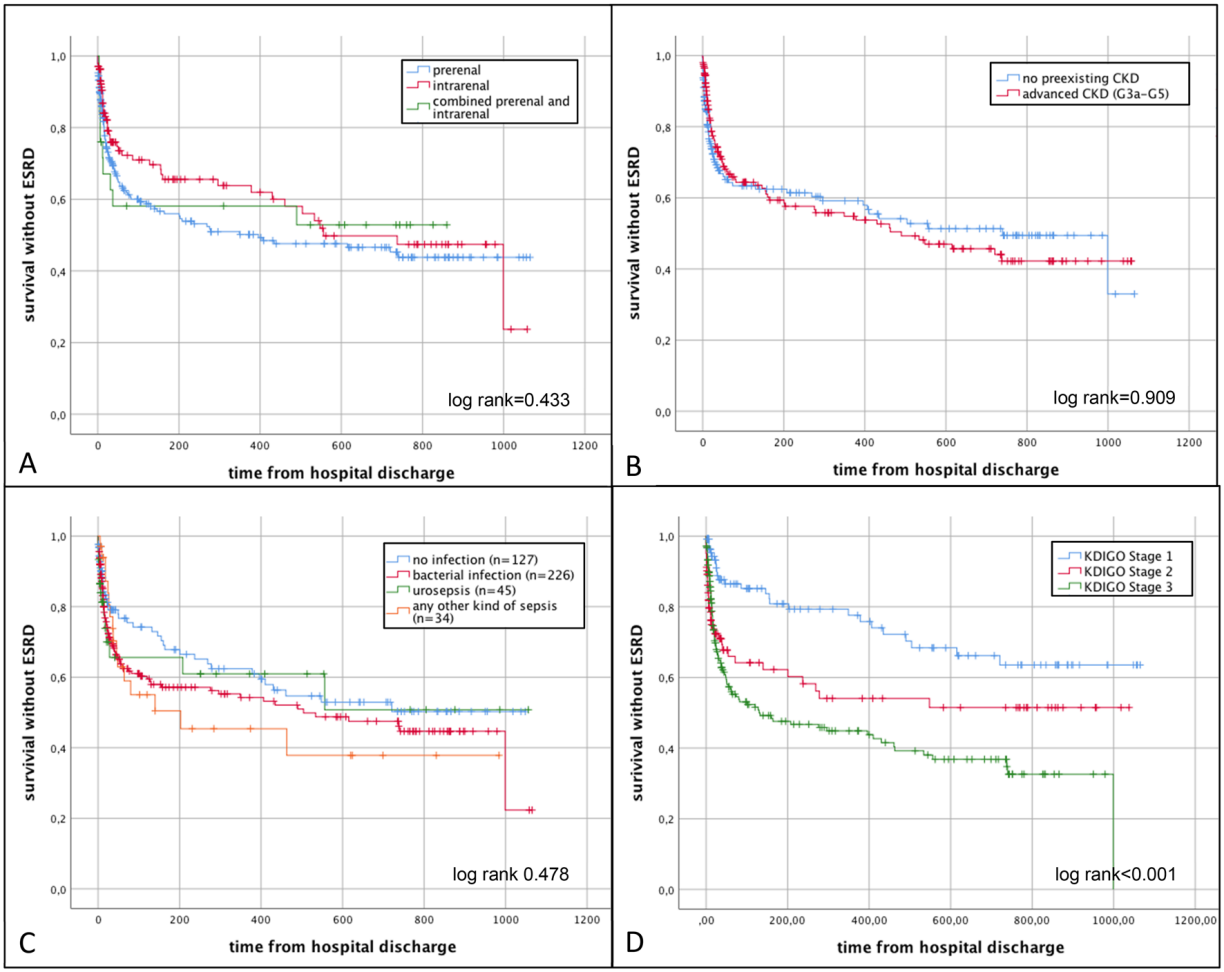
Kaplan–Meier curve for combined endpoint of development of end stage kidney disease (ESKD) or death in patients with acute kidney injury (AKI) with respect to aetiology of AKI (**A**), pre-existence of advanced CKD (**B**), infectious status (**C**) and severity of AKI (**D**)

Overall, 76 patients with AKI died in the hospital (17.6%). Follow-up data were available for 238 of the remaining 356 patients (66.7%) with an average follow-up time of 304 days (range 10–1.065 days). Clinical and laboratory characteristics at baseline did not differ between patients with available follow-up data and those lost to follow-up, except for a higher rate of patients in need of permanent RRT at time of discharge in the follow-up group (Supplemental Table 7). In the course of AKI, serum creatinine usually returned to baseline values observed before the AKI episode. During follow-up, mean serum creatinine in the no-CKD group was 1.12 mg/dl ± 0.41 mg/dl and 2.18 mg/dl ± 0.66 mg/dl in the CKD group, respectively (Fig. 2). Only 13 patients required long-term RRT, and 79 patients died during follow-up time. A Kaplan–Meier curve comparing patients with and without pre-existing advanced CKD regarding the combined endpoint (development of ESKD and death) is shown in Fig. 3B. There was no significant difference in dialysis-free survival between the two groups. However, severity of AKI and treatment in the ICU showed a highly significant impact (log rank < 0.001, Fig. 3D, Supplemental Table 8 and Supplemental Figs. 3 and 4), revealing higher risk of ESKD and mortality.

## Discussion

Acute kidney injury is a very common diagnosis in hospitalised patients, both in medical and surgical departments. Even though criteria for AKI and its grading are precisely defined, the presentation of AKI is highly variable and may affect patients of all ages with or without a complex medical history and both stable as well as critically ill patients. Pathophysiological mechanisms in AKI are also manifold: primary or secondary autoimmune processes, renal involvement in chronic systemic diseases, nephrotoxic medication, hypotension or venous congestion can all affect renal function [15–17]. In most patients, AKI is the result of a multifactorial process [18, 19]. Taking into account the different settings, aetiology and pathophysiological mechanisms, some authors describe AKI more as a syndrome than a diagnosis [20]. Additionally, the large proportion of patients presenting with an “acute on chronic” kidney injury shows the diversity of this diagnosis. Development of AKI and CKD are knowingly in a vicious circle: observational studies demonstrated that AKI leads to newly developed CKD and to progression of pre-existing CKD [21, 22]. On the other hand, CKD patients seem to have an elevated risk of AKI episodes [23]. This connection is also reflected by the results of the present study: more than half of the study population presented with pre-existing CKD, with 15.9% (*n* = 69) of AKI patients being readmitted for another AKI episode within one year. In most cases, creatinine values at time of discharge remained elevated compared to pre-admission values (2.12 mg/dl vs. 1.35 mg/dl), meeting the diagnostic criteria of acute kidney disease (AKD). However, one must consider that patients are often discharged before full renal recovery.

Previous data—mainly investigating AKI in critically ill patients—have identified AKI as an independent risk factor for mortality and morbidity [5, 24]. The correlation of severity of AKI with mortality could be confirmed in the present study cohort, comprising also patients in stable conditions (log rank < 0.001). Van Berendoncks and colleagues showed that mortality following AKI is unrelated to treatment modality; however, the impact of the underlying pathomechanisms and the effect of co-existing or underlying infections on renal and patient outcome have not yet been fully investigated [25]. The present retrospective study tried to fill this gap of knowledge, by analysing AKI patients in a nephrology department in a university setting and investigating the impact of the aetiology and infectious status of AKI. Of interest, patients with urosepsis and AKI experienced the shortest hospital stay (9.7 d, *p* < 0.001) and were least likely to require temporary RRT (17.8%, *p* = 0.003), even though severity of AKI and in-hospital mortality were comparable to other AKI groups. This observation matches our clinical experience in this patient population; patients with urosepsis often present with a fulminant disease onset in urgent need of fluid therapy for volume management and often require vasopressin therapy. However, if treatment is started in time, affected patients show a quick recovery after initiation of antimicrobial and volume therapy [26, 27]. Not surprisingly, patients with other septic foci, predominantly those with pulmonary sepsis, had the longest hospital stay (30 ± 22 days), the most frequent need for RRT (44.1%) and the highest in-hospital mortality (23.5%). Nevertheless, mortality rate in septic patients in the present cohort seems to be rather low when compared to previously published data of a large meta-analysis, revealing an average mortality rate of 32.5% in septic patients in Europe [28]. This might be due to inclusion of only critically ill patients fulfilling sepsis criteria in other studies or since patients in the present study were only treated by nephrologists, assuming a specific goal-directed approach in AKI patients.

Previous data have identified CKD as an independent risk factor for all-cause mortality. Go et al. showed in a longitudinal study the association between level of kidney dysfunction as determined by glomerular filtration rate (GFR) and risk of death. With impaired GFR, mortality increases disproportionately; e.g., CKD stage 5 patients have a 5.9 times higher mortality risk than their peers [29]. In contrast, the present study did not find a significant difference in survival comparing CKD patients to no-CKD patients with AKI (log rank 0.902). Unexpectedly, in-hospital mortality following AKI was lower in patients with pre-existing advanced CKD when compared to mortality in patients without pre-existing CKD (13.4% vs. 21.7%, *p* = 0.032). Khosla et al. made a similar observation in the PICARD study, where in-hospital mortality was lower in AKI patients with pre-existing CKD (31% vs. 40%, *p* = 0.04) [30], while other publications described an elevated risk in patients with “acute on chronic” kidney injury [10, 31]. These findings contribute to the observation by Fiorentino et al. and Peerapornratana and coworkers who showed that recovery from AKI by time of discharge is essential for patients´ long-term outcome: when full recovery can be achieved, mortality is similar to that in non-AKI patients [32, 33]. It is imaginable that patients with CKD have already adapted to the situation of impaired kidney function and that they are less likely to be critically affected by another blow to the kidney, as has been demonstrated with potassium homeostasis in CKD [34, 35]. In concordance with other publications [36], in the present study the percentage of patients requiring permanent RRT following AKI was higher in those with pre-existing CKD vs. those without CKD (13.9% vs. 5.2%, *p* = 0.004).

This study has several strengths and a unique selling point in that it provides representative data on all patients with AKI, not limiting the inclusion criteria to critically ill patients, like in most previous studies. Furthermore, the potential role of the AKI modality and of a pre-existing CKD as well as the impact of infections has been included in the evaluation of possible relevant parameters defining AKI outcome. Including only patients treated in a nephrology department at a university hospital may have improved identification of patients with AKI. Limitations include the retrospective observational study design that makes the results vulnerable for confounding factors. Moreover, a number of patients were lost to follow-up, with a potential impact on long-term outcome results, although baseline characteristics were not different from those included in the follow-up. A prospective study on the subject could strengthen the results.

## Conclusion

In conclusion, the present study shows that in patients with AKI: (1) prerenal and intrarenal causes have no different impact on renal outcome and mortality; (2) infections do not result in worse renal function, more need of RRT or increased mortality, although patients with urosepsis have a significant shorter in-hospital stay; (3) in case of pre-existing CKD the underlying renal disease does not affect the course of AKI and outcome; and (4) in- hospital mortality is lower in AKI patients with pre-existing CKD, but the risk of developing permanent RRT is significantly higher.

### Supplementary Information

Below is the link to the electronic supplementary material.

Supplementary file1 (DOCX 2384 KB)

## Data Availability

The datasets generated during the current study are available from the corresponding author on reasonable request.
