# Supplementary material for: Pre-existing chronic kidney disease, aetiology of acute kidney injury and infection do not affect renal outcome and mortality
Source: J Nephrol. 2023 Oct 3;37(2):391–400. doi: 10.1007/s40620-023-01774-x (PMC11043156; doi:10.1007/s40620-023-01774-x)
Supplement: Supplementary file 1 — Supplementary file1 (DOCX 2384 KB) [file 40620_2023_1774_MOESM1_ESM.docx]

**Supplemental table 1:** sample size collection

**F tests** - ANOVA: Repeated measures, within-between interaction

**Analysis:** A priori: Compute required sample size

**Input:** Effect size f = 0,15

α err prob = 0,05

Power (1-β err prob) = 0,95

Number of groups = 4

Number of measurements = 3

Corr among rep measures = 0,5

Nonsphericity correction ε = 1

**Output:** Noncentrality parameter λ = 21,6000000

Critical F = 2,1276806

Numerator df = 6,0000000

Denominator df = 312

Total sample size = 160

Actual power = 0,9531741

**F tests** - ANOVA: Repeated measures, within-between interaction

**Analysis:** A priori: Compute required sample size

**Input:** Effect size f = 0,15

α err prob = 0,05

Power (1-β err prob) = 0,95

Number of groups = 4

Number of measurements = 3

Corr among rep measures = 0,5

Nonsphericity correction ε = 1

**Output:** Noncentrality parameter λ = 21,6000000

Critical F = 2,1276806

Numerator df = 6,0000000

Denominator df = 312

Total sample size = 160

Actual power = 0,9531741


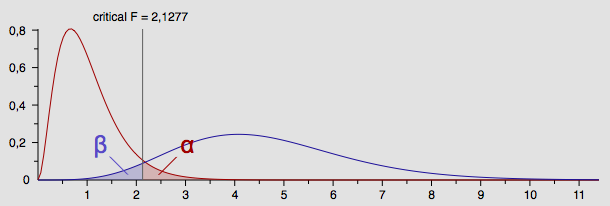


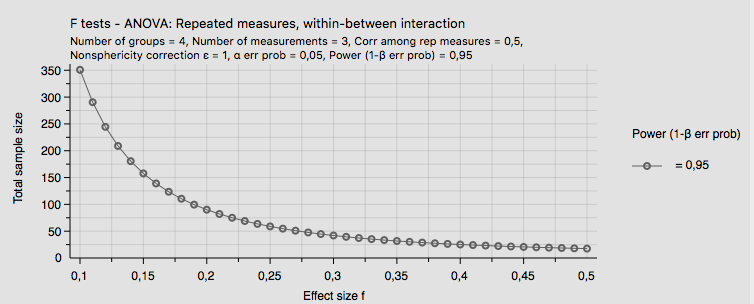


**Supplemental table 2**: STROBE statement
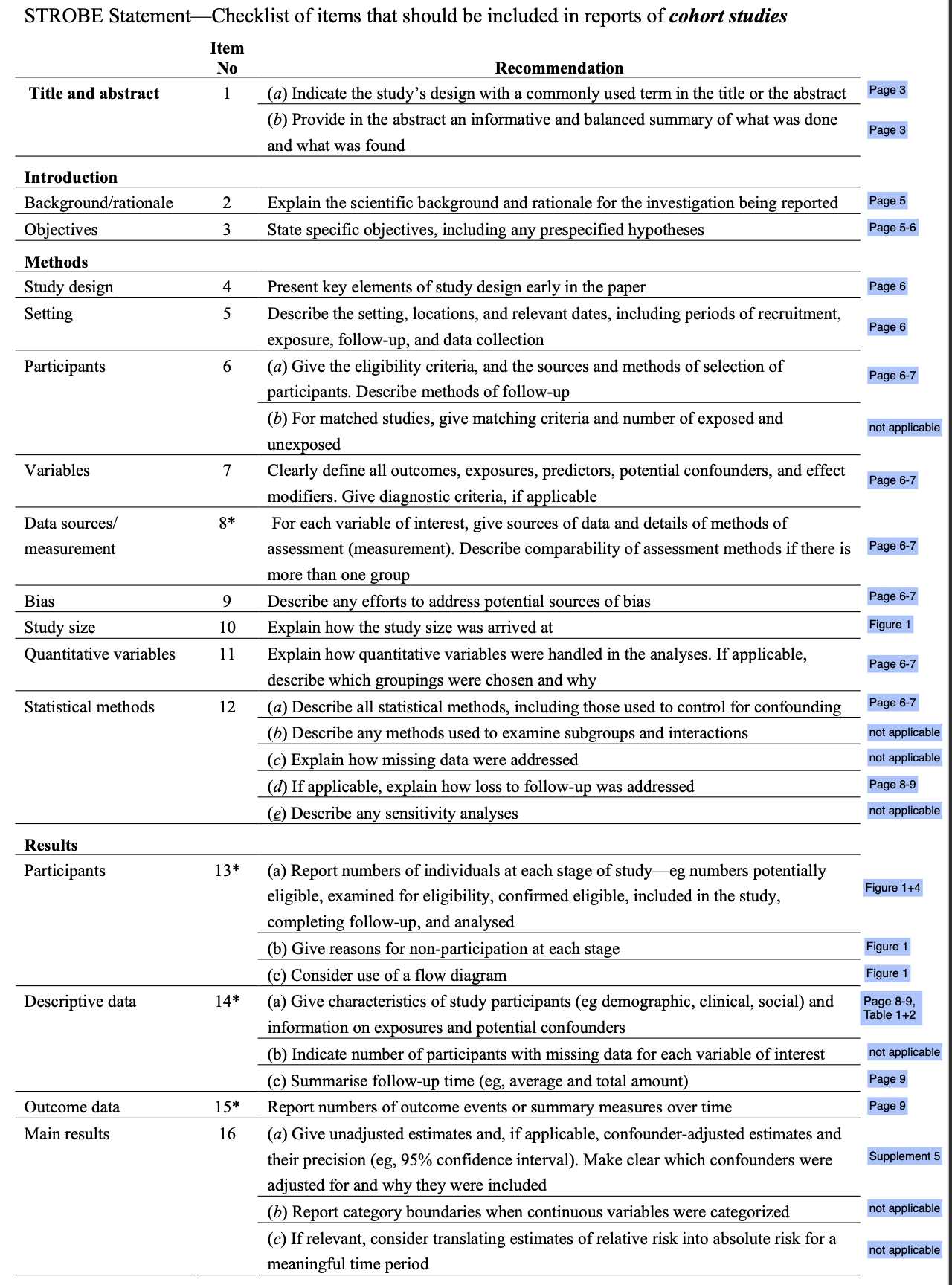

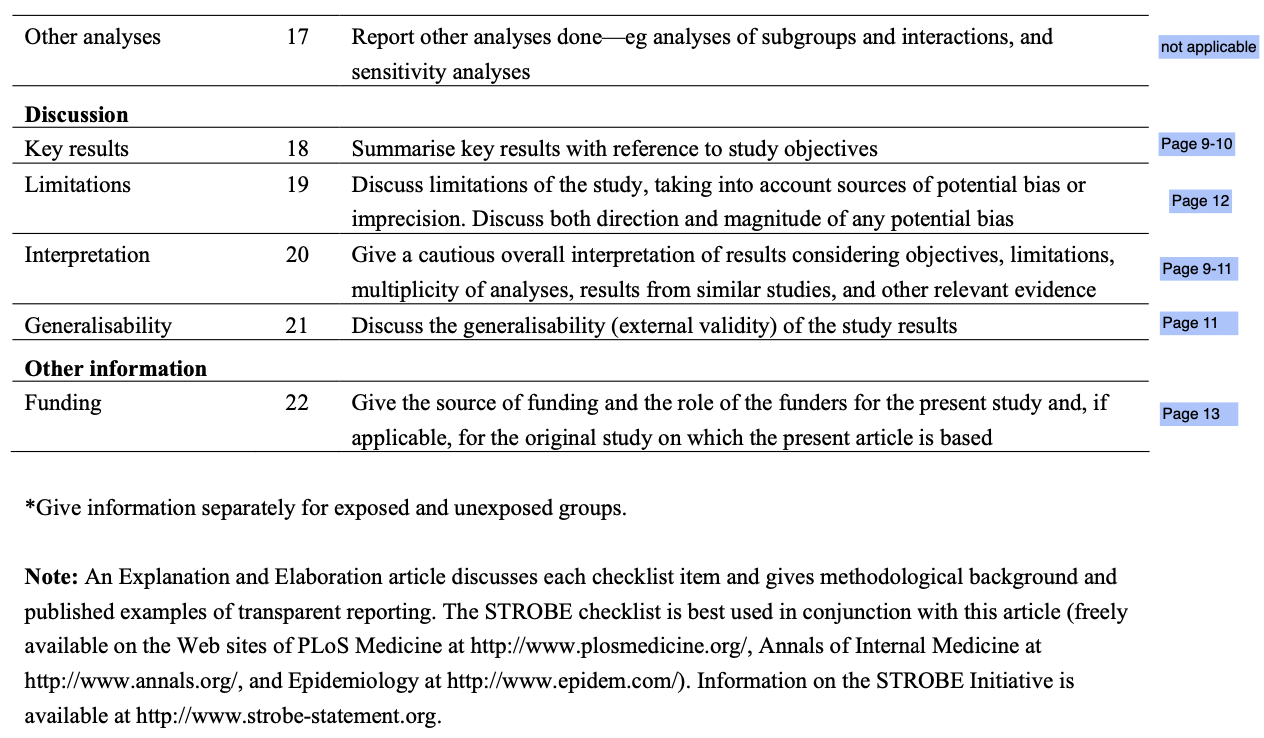


**Supplemental figure 1:** Underlying renal disease in patients with pre-existing chronic kidney disease (n=241)

**Supplemental table 3:** Baseline characteristics in patients with chronic kidney disease due to underlying renal disease.

|  | | | **Hypertensive nephropathy (n=42)** | | | | | **Diabetic nephropathy (n=13)** | | **Hypertensive**  **diabetic nephropathy (n=32)** | | | | | | | | **Vasculitis (n=20)** | | | | **IgA-Nephropathy (n=12)** | | | | | | **Cardiorenal/**  **hepatorenal**  **Syndrome (n=29)** | | | | | | | **P value** | | | | | | |  |  |  |  |  |
| --- | --- | --- | --- | --- | --- | --- | --- | --- | --- | --- | --- | --- | --- | --- | --- | --- | --- | --- | --- | --- | --- | --- | --- | --- | --- | --- | --- | --- | --- | --- | --- | --- | --- | --- | --- | --- | --- | --- | --- | --- | --- | --- | --- | --- | --- | --- |
| **Age (years)** | | | 77.8 | | | | 68.0 | | | | 77.5 | | | | | | 66.7 | | | | 49.0 | | | | | | | | 73.7 | | | | | | | **<.001^a^** | | | | | | |  |  |  |  |
| **Male** | | | 25 (59.5%) | | | | 7 (53.8%) | | | | 26 (81.3%) | | | | | | 10 (50.0%) | | | | 11 (91.7%) | | | | | | | | 17 (58.6%) | | | | | | | **.04^b^** | | | | | | |  |  |  |  |
| **Duration of hospital stay (d)** | | | 14.7 | | | | 25.3 | | | | 11.7 | | | | | | 13.1 | | | | 9.0 | | | | | | | | 15.1 | | | | | | | **.08^c^** | | | | | | |  |  |  |  |
| **Temporary dialysis** | | | 10 (23.8%) | | | | 6 (46.2%) | | | | 10 (31.3%) | | | | | | 2 (10.0%) | | | | 1 (8.3%) | | | | | | | | 8 (27.6%) | | | | | | | .15 | | | | | | |  |  |  |  |
| **Discharge with dialysis** | | | 6 (14.3%) | | | | 2 (15.4%) | | | | 7 (21.9%) | | | | | | 1 (5.0%) | | | | 0 (0.0%) | | | | | | | | 4 (13.8%) | | | | | | | .41 | | | | | | |  |  |  |  |
| **In-hospital mortality** | | | 4 (9.5%) | | | | 2 (15.4%) | | | | 3 (9.4%) | | | | | | 1 (5.0%) | | | | 0 (0.0%) | | | | | | | | 5 (17.2%) | | | | | | | .56 | | | | | | |  |  |  |  |
| **Severity AKI** | | |  | | | | |  | |  | | | | | | | | |  | | | | |  | | | | | | |  | | | | | | |  | | | | | | |  |  |
|  |  | AKIN I | 10 (23.8%) | | | | | 4 (30.8%) | | | | | 15 (46.9%) | | 8 (40.0%) | | | | | | | | 1 (8.3%) | | | | 10 (34.5%) | | | | | | | | .14 | | | | | |  |  |  |  |  |  |
|  |  | AKIN II | 11 (26.2%) | | | | | 4 (30.8%) | | | | | 6 (18.3%) | | 4 (20.0%) | | | | | | | | 5 (41.7%) | | | | 5 (17.2%) | | | | | | | | .57 | | | | | |  |  |  |  |  |  |
|  |  | AKIN III | 21 (50.0%) | | | | | 5 (38.5%) | | | | | 11 (34.4%) | | 8 (40.0%) | | | | | | | | 6 (50.0%) | | | | 14 (48.3%) | | | | | | | | .78 | | | | | |  |  |  |  |  |  |
| **Pre-existing CKD** | | |  | | | | |  | |  | | | | | | | | |  | | | | |  | | | | | | |  | | | | | | |  | | | | | | | |  |
|  |  | KDIGO G1 | | 2 (4.8%) | | | | 1 (8.3%) | | | | | 1 (3.3%) | | | 0 (0.0%) | | | | | | | 0 (0.0%) | | | | | | | 1 (3.4%) | | | | | | | .82 | | | | | | |  |  |  |
|  |  | KDGIO G2 | | 2 (4.8%) | | | | 1 (8.3%) | | | | | 3 (10.0%) | | | 0 (0.0%) | | | | | | | 2 (16.7%) | | | | | | | 0 (0.0%) | | | | | | | .21 | | | | | | |  |  |  |
|  |  | KDGIO G3a | | 5 (11.9%) | | | | 0 (0.0%) | | | | | 6 (20.0%) | | | 1 (5.0%) | | | | | | | 0 (0.0%) | | | | | | | 5 (17.2%) | | | | | | | .23 | | | | | | |  |  |  |
|  |  | KDIGO G3b | | 8 (19.0%) | | | | 0 (0.0%) | | | | | 3 (10.0%) | | | 3 (15.0%) | | | | | | | 1 (8.3%) | | | | | | | 4 (13.8%) | | | | | | | .60 | | | | | | |  |  |  |
|  |  | KDIGO G4 | | 7 (16.7%) | | | | 4 (33.3%) | | | | | 5 (16.4%) | | | 3 (15.0%) | | | | | | | 3 (25.0%) | | | | | | | 5 (17.2%) | | | | | | | .79 | | | | | | |  |  |  |
|  |  | KDIGO G5 | | 3 (7.1%) | | | | 2 (16.7%) | | | | | 1 (3.3%) | | | 3 (15.0%) | | | | | | | 1 (8.3%) | | | | | | | 2 (6.9%) | | | | | | | .64 | | | | | | |  |  |  |
| **Comorbidities** | | |  | | | | |  | |  | | | | | | | | |  | | | | |  | | | | | | |  | | | | | | |  | | | | | | | |  |
|  |  | Hypertension | | 43 (100%) | | | | 10 (83.3%) | | | | | 20 (62.5%) | | | 14 (70.0%) | | | | | | | 10 (83.3%) | | | 21 (75.0%) | | | | | | | **<.001^d^** | | | | | | |  |  |  |  |  |  |  |
|  |  | Diabetes mellitus | | 10 (23.8%) | | | | 13 (100%) | | | | | 30 (93.8%) | | | 6 (30.0%) | | | | | | | 1 (8.3%) | | | 10 (34.5%) | | | | | | | **<.001^e^** | | | | | | |  |  |  |  |  |  |  |
|  |  | Chronic heart failure | | 23 (57.1%) | | | | 7 (58.3%) | | | | | 13 (40.6%) | | | 11 (55.0%) | | | | | | | 7 (58.3%) | | | 14 (50.0%) | | | | | | | .76 | | | | | | |  |  |  |  |  |  |  |
| **Laboratory parameters (mg/dl)** | | | | | |  | | |  | | | | |  | | | | | |  | | | | |  | | | | | | |  | | | | | | |  | | | | | | | |
|  | First creatinine | | | | 4.21 | | | 3.16 | | | | 4.67 | | | 2.79 | | | | | | | 4.24 | | | | 2.98 | | | | | | | | .13 | | | | | |  |  |  |  |  |  |  |
|  | Maximum creatinine | | | | 4.66 | | | 4.34 | | | | 5.2 | | | 3.28 | | | | | | | 4.53 | | | | 3.67 | | | | | | | | .25 | | | | | |  |  |  |  |  |  |  |
|  | Last creatinine * | | | | 2.12 | | | 1.99 | | | | 2.91 | | | 2.37 | | | | | | | 2.84 | | | | 2.07 | | | | | | | | .23 | | | | | |  |  |  |  |  |  |  |

AKI acute kidney injury; CKD chronic kidney disease

*Discharged patients still in need of renal replacement therapy are excluded

post-hoc comparisons between groups (Tukey´s test): ^a^ IgA-Nephropathy < all other groups; ^b^ IgA-Nephropathy > all other groups;

^c^ diabetic nephropathy > all other groups; ^d^ hypertensive nephropathy > all other groups; ^e^ diabetic nephropathy > all other groups

**Supplemental table 4:** Baseline characteristics according to aetiology of AKI

|  | | prerenal AKI (n=252) | | Intrinsic AKI (n=136) | Combined prerenal and intrinsic AKI (n=44) | p value |
| --- | --- | --- | --- | --- | --- | --- |
| **Age** | | 69.9 | | 68.2 | 72.1 | .32 |
| **Male** | | 164 | | 76 | 31 | .06 |
| **Duration of hospital stay (d)** | | 15.6 | | 13.9 | 14.2 | .56 |
| **Temporary dialysis** | | 70 | | 23 | 10 | .06 |
| **Discharge with dialysis** | | 28 | | 9 | 2 | .20 |
| **In-hospital mortality** | | 53 (21.0%) | | 17 (12.5%) | 6 (13.6%) | .08 |
| **Severity of AKI** | |  | |  |  |  |
|  | AKI I | 68 (26.9%) | | 43 (31.6%) | 9 (20.4%) | .38 |
|  | AKI II | 62 (24.6%) | | 30 (22.0%) | 11 (25.0%) | .85 |
|  | AKI III | 122 (48.4%) | | 63 (46.3%) | 24 (54.5%) | .63 |
| **Pre-existing CKD** | |  | |  |  |  |
|  | KDIGO G1 | | 5 (2.0%) | 2 (1.5%) | 1 (2.4%) | .09 |
|  | KDIGO G2 | | 14 (5.6%) | 9 (6.6%) | 3 (7.3%) | .87 |
|  | KDIGO G3a | | 22 (8.7%) | 11 (8.1%) | 6 (14.3%) | .43 |
|  | KDIGO G3b | | 31 (12.3%) | 22 (16.2%) | 5 (11.9%) | .33 |
|  | KDIGO G4 | | 40 (15.9%) | 19 (14.0%) | 3 (7.1%) | .34 |
|  | KDIGO G5 | | 23 (9.1%) | 13 (9.6%) | 7 (16.7%) | .29 |
| **Comorbidities** | |  | |  |  |  |
|  | Hypertension | 169 (67.1%) | | 93 (68.4%) | 32 (76.2%) | .44 |
|  | Diabetes mellitus | 86 (34.1%) | | 47 (34.6%) | 15 (35.7%) | .99 |
|  | Chronic heart failure | 122 (48.4%) | | 63 (46.3%) | 22 (50.0%) | .72 |
| **Laboratory parameters** | |  | |  |  |  |
|  | Pre-admission creatinine | 1.36 | | 1.37 | 1.19 | .72 |
|  | First creatinine | 2.95 | | 3.07 | 4.18 | **.02^a^** |
|  | Maximum creatinine | 3.65 | | 3.98 | 4.72 | .07 |
|  | Last creatinine prior to dismissal* | 2.12 | | 2.18 | 1.94 | .71 |

AKI acute kidney injury; CKD chronic kidney disease

*Discharged patients still in need of renal replacement therapy are excluded

post-hoc comparisons between groups (Tukey´s test):

^a^combined prerenal and intrinsic AKI > prerenal AKI

**Supplemental table 5:** Multivariable logistic regression model of potential risk factors for the development of severe AKI (KDIGO 3), intra-hospital death and permanent need for RRT.

|  | **Risk of development of severe AKI** | | **Risk of intra-hospital death** | | **Risk of discharge in need of RRT** | |  |
| --- | --- | --- | --- | --- | --- | --- | --- |
|  | **p-value** | **Adjusted odds-ratio (OR, 95% CI)** | **p-value** | **Adjusted odds-ratio (OR, 95% CI)** | **p-value** | **Adjusted odds-ratio (OR, 95% CI)** | |
| Age | .61 | 0.99 (0.98-1.01) | .96 | 0.99 (0.95-1.04) | .94 | 0.99 (0.96-1.03) | |
| Sex | .52 | 0.84 (0.51-1.41) | .61 | 0.68 (0.15-3.02) | .35 | 1.59 (0.59-4.29) | |
| Sodium level* | .24 | 0.98 (0.94-1.01) | .46 | 0.96 (0.88-1.06) | .48 | 1.03 (0.95-1.11) | |
| Proteinuria* | .69 | 1.09 (0.69-1.70) | .57 | 0.46 (0.03-6.87) | **.004** | **1.91 (1.23-2.98)** | |
| α1-microglobulin* | .43 | 0.99 (0.99-1.01) | .76 | 1.00 (0.98-1.01) | .69 | 1.00 (0.99-1.01) | |
| albuminuria* | .44 | 1.00 (0.99-1.00) | .93 | 1.00 (0.99-1.01) | .017 | 0.99 (0.99-1.00) | |
| Intake of loop diuretics* | .26 | 0.75 (0.45-1.24) | .22 | 0.39 (0.08-1.76) | .44 | 1.48 (0.53-4.09) | |

AKI, acute kidney injury; RRT, renal replacement therapy

*values determined at time of admission

**Supplemental table 6**: Cox regression analysis analysing prognostic impact of type of AKI, pre-existent CKD and infectious status, unadjusted and adjusted to age.

|  | | **Patients reaching combined endpoint*** | **Hazard ratio (95% CI)** | |
| --- | --- | --- | --- | --- |
|  | |  | **unadjusted** | **adjusted for age** |
| **Type of AKI** | |  |  |  |
|  | Prerenal | 103/252 | 1.26 (0.73-2.16) | 1.20 (0.65-2.19) |
|  | Intrarenal | 46/136 | 0.99 (0.56-1.80) | 0.96 (0.49-1.85) |
|  | Combined pre- and intrarenal | 15/44 | 0.85 (0.67-1.08) | 0.80 (0.77-1.21) |
| **Pre-existent CKD** | |  |  |  |
|  | No CKD | 72/197 | 0.97 (0.71-1.32) | 0.82 (0.57-1.18) |
|  | Pre-existent CKD | 92/235 | 1.07 (0.78-1.45) | 1.27 (0.88-1.83) |
| **Infectious status** | |  |  |  |
|  | No infection | 43/127 | 0.71 (0.40-1.26) | 0.61 (0.31-1.18) |
|  | Bacterial infection | 92/226 | 0.92 (0.54-1.58) | 0.81 (0.43-1.51) |
|  | Sepsis | 30/79 | 0.89 (0.42-1.78) | 0.73 (0.32-1.66) |

*combined endpoint: death or development of ESKD

**Supplemental table 7**: Baseline characteristics comparing patients with available follow up data to patients lost to follow up.

|  | | Patients lost to follow up (n=118) | | Patients with follow up data (n=238) | p value |
| --- | --- | --- | --- | --- | --- |
| **Age** | | 70.1 | | 68.4 | .52 |
| **Male** | | 68 (57.6%) | | 154 (64.7) | .17 |
| **Duration of hospital stay (d)** | | 13.7 | | 16.2 | .28 |
| **Temporary dialysis** | | 17 (14.4%) | | 50 (21.0%) | .30 |
| **Discharge with dialysis** | | 2 (1.6%) | | 29 (12.2%) | **.004** |
| **Severity of AKI** | |  | |  |  |
|  | AKI I | 28 (23.7%) | | 76 (31.9%) | .21 |
|  | AKI II | 28 (23.7%) | | 55 (23.1%) | .84 |
|  | AKI III | 62 (52.5%) | | 107 (44.9%) | .24 |
| **Pre-existing CKD** | |  | |  |  |
|  | KDIGO G1 | | 0 (0.0%) | 7 (2.9%) | .16 |
|  | KDIGO G2 | | 7 (5.9%) | 15 (6.3%) | .95 |
|  | KDIGO G3a | | 7 (5.9%) | 26 (10.9%) | .29 |
|  | KDIGO G3b | | 15 (12.7%) | 34 (14.3%) | .06 |
|  | KDIGO G4 | | 16 (13.6%) | 36 (15.1%) | .84 |
|  | KDIGO G5 | | 15 (12.7%) | 22 (9.2%) | .56 |
| **Comorbidities** | |  | |  |  |
|  | Hypertension | 76 (64.4%) | | 167 (70.1%) | .27 |
|  | Diabetes mellitus | 41 (34.7%) | | 90 (37.8%) | .64 |
|  | Chronic heart failure | 60 (50.8%) | | 111 (49.6%) | .52 |
| **Laboratory parameters** | |  | |  |  |
|  | Pre-admission creatinine | 1.20 | | 1.41 | .09 |
|  | First creatinine | 3.42 | | 3.14 | .61 |
|  | Maximum creatinine | 3.84 | | 3.88 | .77 |
|  | Last creatinine prior to dismissal* | 1.78 | | 2.11 | .16 |

*Discharged patients still in need of renal replacement therapy are excluded

**Supplemental table 8**: Baseline characteristics in patients with and without treatment in ICU

|  | | Treatment in ICU (n=173) | | No treatment in ICU (n=259) | p value |
| --- | --- | --- | --- | --- | --- |
| **Age** | | 68.4 | | 70.4 | .18 |
| **Male** | | 120 (69.4%) | | 152 (58.5%) | .24 |
| **Duration of hospital stay (d)** | | 17.9 | | 13.0 | **.001** |
| **Temporary dialysis** | | 57 (32.9%) | | 46 (17.8%) | **<.001** |
| **Discharge with dialysis** | | 19 (11.0%) | | 20 (7.7%) | .25 |
| **In-hospital mortality** | | 56 (32.4%) | | 20 (7.7%) | **<.001** |
| **Severity of AKI** | |  | |  |  |
|  | AKI I | 37 (21.4%) | | 73 (32.0%) | .81 |
|  | AKI II | 46 (26.6%) | | 61 (21.6%) | .97 |
|  | AKI III | 90 (52.0%) | | 125 (46.3%) | .86 |
| **Pre-existing CKD** | |  | |  |  |
|  | KDIGO G1 | | 3 (1.7%) | 5 (1.9%) | .87 |
|  | KDIGO G2 | | 10 (5.8%) | 16 (6.2%) | .84 |
|  | KDIGO G3a | | 16 (9.2%) | 8.9 (9.0%) | .92 |
|  | KDIGO G3b | | 22 (12.7%) | 36 (13.9%) | .69 |
|  | KDIGO G4 | | 24 (13.9%) | 38 (14.7%) | .78 |
|  | KDIGO G5 | | 19 (9.8%) | 26 (10.0%) | .91 |
| **Comorbidities** | |  | |  |  |
|  | Hypertension | 117 (67.6%) | | 179 (69.1%) | .69 |
|  | Diabetes mellitus | 52 (30.0%) | | 97 (37.5%) | .11 |
|  | Chronic heart failure | 82 (47.4%) | | 127 (49.0%) | .70 |
| **Laboratory parameters** | |  | |  |  |
|  | Pre-admission creatinine | 1.22 | | 1.42 | .10 |
|  | First creatinine | 2.76 | | 3.35 | .32 |
|  | Maximum creatinine | 3.78 | | 3.92 | .62 |
|  | Last creatinine prior to dismissal* | 2.07 | | 2.16 | .60 |

**Supplemental figure 3**: In-hospital mortality rate according to AKI stage. There can be a positive correlation between severity of AKI and mortality rate (r=0.156, p<0.01).


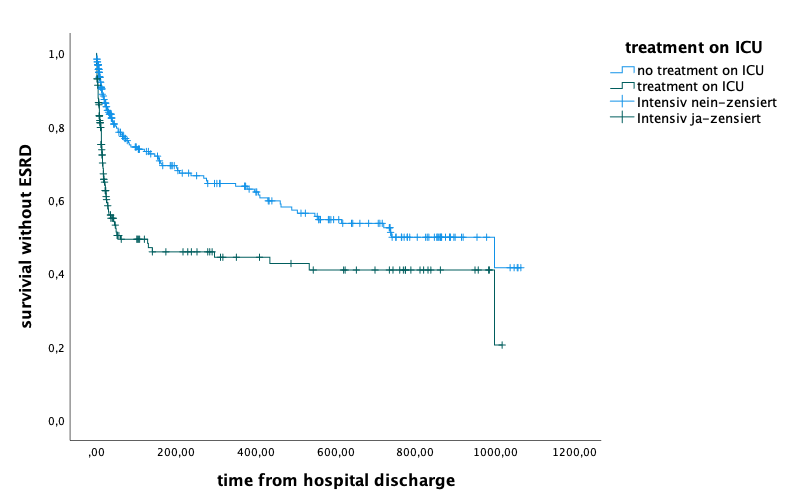


log rank<0.001

**Supplemental figure 4**: Kaplan-Meier curve for combined endpoint of development of end-stage kidney disease (ESKD) or death in patients comparing patients with and without temporary treatment in ICU.
